# Opioid-induced hyperalgesia in chronic pain patients and the mitigating effects of gabapentin

**DOI:** 10.3389/fphar.2015.00104

**Published:** 2015-05-27

**Authors:** Nicoleta Stoicea, Daric Russell, Greg Weidner, Michael Durda, Nicholas C. Joseph, Jeffrey Yu, Sergio D. Bergese

**Affiliations:** ^1^Department of Anesthesiology, The Ohio State University Wexner Medical CenterColumbus, OH, USA; ^2^Department of Neuroscience, The Ohio State UniversityColumbus, OH, USA; ^3^Medical School, The Ohio State University College of MedicineColumbus, OH, USA; ^4^Department of Neurological Surgery, The Ohio State University Wexner Medical CenterColumbus, OH, USA

**Keywords:** gabapentin, opiods, opiod-induced hyperalgesia, allodynia, NMDA, VGCCs, neuropathic pain

## Abstract

Chronic pain patients receiving opioid drugs are at risk for opioid-induced hyperalgesia (OIH), wherein opioid pain medication leads to a paradoxical pain state. OIH involves central sensitization of primary and secondary afferent neurons in the dorsal horn and dorsal root ganglion, similar to neuropathic pain. Gabapentin, a gamma-aminobutyric acid (GABA) analog anticonvulsant used to treat neuropathic pain, has been shown in animal models to reduce fentanyl hyperalgesia without compromising analgesic effect. Chronic pain patients have also exhibited lower opioid consumption and improved pain response when given gabapentin. However, few human studies investigating gabapentin use in OIH have been performed in recent years. In this review, we discuss the potential mechanisms that underlie OIH and provide a critical overview of interventional therapeutic strategies, especially the clinically-successful drug gabapentin, which may reduce OIH.

## Introduction

Effective attenuation of pain is of the utmost importance in patient care. Opioid medications are commonly used in perioperative pain management in addition to providing relief for back (Chu et al., 2006) and burn patients (Holtman and Jellish, 2012). Paradoxically, increased use of these medications has revealed side effects including opioid dependence, tolerance, and opioid-induced hyperalgesia (OIH). OIH is characterized by a paradoxical state of heightened pain sensation in which both pain threshold and pain tolerance decrease (Kim et al., 2014). Moreover, this effect is seen not only in the chronic user but also surgical populations receiving opioids intraoperatively (Ballantyne, 2006; Akbari, 2012; Lee et al., 2013a,b).

OIH is similar to opioid tolerance in that opioid drugs exhibit diminished efficacy with time, such that increasing dosage is required to provide a consistent level of analgesic effect (Chu et al., 2006; Akbari, 2012). However, OIH differs from opioid tolerance in that dose increases are accompanied by an increase in pain sensitivity and higher pain scores (Ballantyne, 2006; Compton et al., 2010; Akbari, 2012). OIH is also accompanied by allodynia, pain caused by a stimulus that does not normally provoke pain (Fishbain et al., 2009; Motoc et al., 2011; Bravo-Hernández et al., 2012). It is thought that the analgesic and hyperalgesic effects of opioids exist at the same time, but the analgesic effects normally predominate, masking the hyperalgesia that is present (Ballantyne, 2006). While the precise mechanism of OIH is still being researched, the interplay between n-methyl-D-aspartate (NMDA) and μ glutaminergic receptors is a particularly probable mechanism, substantiated by these receptors' proximity to one another on primary afferent glutamate neurons within the mesencephalic periaqueductal gray region—a major pain pathway (Rodríguez-Muñoz et al., 2012).

Human studies have revealed that several commonly used opioid drugs including fentanyl, remifentanil, and morphine have the ability to induce OIH (Lenz et al., 2011; Motoc et al., 2011; Bravo-Hernández et al., 2012; Raffa and Pergolizzi, 2013). Remifentanil induces hyperalgesia and opioid tolerance in a dose-dependent manner (Richebé et al., 2011). An infusion of remifentanil (0.1 μg/kg/min) induced acute opioid tolerance in rat models subjected to the cold pressor (CP) test, where a rate of 0.08 μg/kg/min prevented tolerance from developing (Kim et al., 2014). Human patients receiving higher intraoperative doses of remifentanil (0.3 ± 0.2 and 0.4 μg/kg/min) exhibited decreased time until rescue opioids were required as well as increased opioid use within the 24–48-h postoperative period (Joly et al., 2005; Kim et al., 2014). In particular, the study saw higher visual analog scale scores in the remifentanil group as compared to the placebo group; however this difference was no longer significant 2–24 h after surgery. NMDA-antagonists, α_2_-agonists, non-steroidal anti-inflammatory drugs, and GABA analogs, along with opioid rotation have previously been shown to lessen OIH (Koppert and Schmelz, 2007; Vorobeychik et al., 2008; Akbari, 2012; Holtman and Jellish, 2012; Pasero and McCaffery, 2012). In this review, we focus on gabapentin as a method for treatment of remifentanil and fentanyl induced-hyperalgesia.

## Suggested mechanisms of opioid induced hyperalgesia

Central pain sensitization, NMDA receptor activity, and spinal dynorphin release have all been implicated as the source of OIH (Daeninck and Bruera, 1999; Gardell et al., 2002; Koppert and Schmelz, 2007; Gupta et al., 2011). OIH is comparable to neuropathic pain in both its neuro-inflammatory qualities and central sensitization processes. In neuropathic pain and OIH alike, there is ascending central hyperexcitability and diminished descending supraspinal inhibition, causing increased sensitivity to nociceptive inputs (Compton et al., 2010).

### NMDA and μ-opioid receptor interactions

NMDA receptor activity has quickly gathered attention as one of the mechanisms involved in the propagation of hyperalgesia. As μ-opioid receptors are bound, nitric oxide (NO)-mediated NMDAR potentiation leads via Src-regulated recruitment of PKC and Gα subunits, to an increase in NMDA Ca^2+^ channels (Garzon et al., 2008; Rodríguez-Muñoz et al., 2012). This NMDAR increase leads in turn to an upregulation of the NO synthase cascade and negative functional regulation of morphine algesia, as well as to protein kinase C-mediated phosphorylation of opioid receptors (Koppert and Schmelz, 2007; da Cunha Leal et al., 2010; Rodríguez-Muñoz et al., 2012). Increased NMDAR activation may also downregulate glutamate reuptake mechanisms, leading to central sensitization (Vorobeychik et al., 2008; da Cunha Leal et al., 2010; Lenz et al., 2011; Tompkins and Campbell, 2011; Wilson et al., 2011; Holtman and Jellish, 2012; Juba et al., 2013).

**Figure 1:**
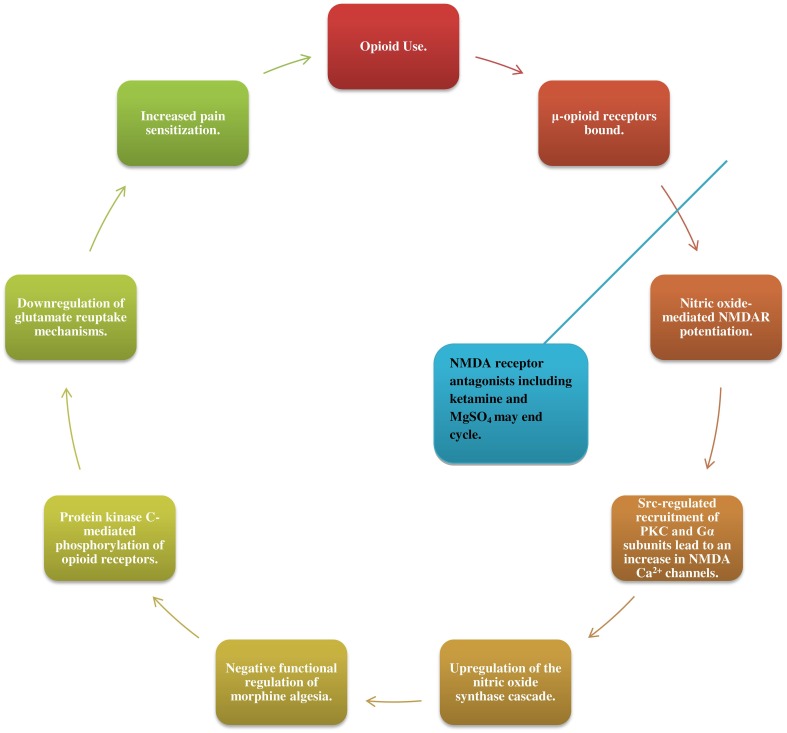
**Opioids bind to μ-opioid receptors triggering nitric oxide-mediated potentiation of N-methyl-D-aspartate receptors (NMDAR) and lead, via SRC-regulated recruitment of PKC and Gα subunits, to an increase in NMDA Ca^2+^ channels**. This enhanced NMDAR activity leads in turn to an upregulation of the nitric oxide synthase cascade-a negative functional regulator of morphine algesia, as well as to protein kinase C-mediated phosphorylation of opioid receptors and downregulation of glutamate reuptake mechanisms. Together, these changes bring about central sensitization to pain and an increased need for opioid medications, sustaining the cycle. Patients receiving NMDAR antagonists such as ketamine and MgSO_4_ alongside opioids exhibited recovery of opioid analgesic effect.

Patients receiving NMDA receptor antagonists including ketamine (da Cunha Leal et al., 2010) and MgSO_4_ alongside opioids have exhibited recovery of opioid analgesic effect, further substantiating these proposed mechanisms (Figure 1) (Daeninck and Bruera, 1999; Gupta et al., 2011; Colvin and Fallon, 2010; Pasero and McCaffery, 2012; Lee et al., 2013a).

However, other classical studies have shown nitric oxide to inhibit rather than potentiate NMDARs in cortical neurons. The proposed mechanism for this NO inhibition involves reaction with the GluN2A subunit C399 and two cysteine groups (Takahashi et al., 2007). These seemingly disparate results are partially explained in studies of the frequency at which presynaptic neurons are stimulated. Frequent neuronal stimulation leads to potentiation of the NMDA receptors while a lesser frequency leads to opposing inhibition and nitrosation of NMDA receptors. Such a hypothesis demonstrates both the complexity and sensitivity of NMDAR-μ-opioid receptor interactions within the opioid user (Garthwaite and Boulton, 1995).

### Spinal dynorphin release

Opioid-associated pain may also result from neuroplastic changes in the rostral ventromedial medulla (RVM). A study by Gardell et al. performed a microinjection of lidocaine into the RVM or bilateral lesion of the dorsolateral funiculus. The injection abolished tactile and thermal hypersensitivity, indicating the RVM modulates nociceptive input. The same study correlated chronic morphine exposure with upregulation of immune reactivity for calcintonin gene-related peptides and spinal dynorphin (Gardell et al., 2002). The role of spinal dynorphin in attenuating pain is not yet clear, however, a recent study correlates activation of spinal bradykinin receptors by elevated spinal dynorphin with maintenance of inflammatory hyperalgesia (Bannister et al., 2014).

## Proposed gabapentin mitigating mechanisms

Gabapentin, a GABA analog commonly used as an anticonvulsant, has also been used in the treatment of neuropathic pain caused by diabetes, chronic illness, and a wide variety of surgeries, to great effect. When given prior to and after surgery, gabapentin has been shown to significantly decrease postoperative pain scores. Gabapentin has also proven effective against postoperative nausea and vomiting within the first 24 h when given alongside dexamethasone, and significantly reduced the rate of postoperative sore throat in another study. Perioperative use of gabapentin was also correlated with a reduction in opioid consumption and the associated side effects while contributing few side effects of its own, namely nausea and dizziness. The side effects of gabapentin were seen most commonly in the chronic user, and were of concern mainly for elderly populations where such side effects might increase the risk of falling. Gabapentin's side effects were not increased in opioid-abusing patients on concurrent methadone use and at risk for OIH (Compton et al., 2010; Chang et al., 2014). Such a drug which effectively and more safely attenuates nociceptive pain is of great interest to those searching for an effective method for treating and reducing OIH.

Although it is a GABA analog, gabapentin does not bind to GABA_A_ or GABA_B_ receptors, block GABA uptake or metabolism, or have any direct action on the GABAnergic system (Chang et al., 2014). While several studies have demonstrated the efficacy of gabapentin in reducing postoperative hyperalgesia and allodynia, the exact mechanism by which it alleviates pain is poorly understood. Suggested pharmacologic mechanisms involve its effect on two pathways: α-2δ Ca^2+^ channels, and the interleukin-10-Heme Oxygenase-1 signaling pathway, both of which play distinct roles in the processing of pain (Zoidis et al., 2005; Chang et al., 2014; Bao et al., 2014; Suto et al., 2014)^1^.

### Voltage gated calcium channels

Central sensitization resulting from increased activity of afferent nociceptive neurons has been shown to be a key contributing factor in heightened pain sensitivity. Gabapentin reduces central sensitization through attenuating lesion-induced hyperexcitability of posterior horn neurons. The drug binds postsynaptically to α-2δ subunits of dorsal horn neurons' voltage-gated calcium channels (VGCCs) which are upregulated in neuropathic pain states (Chang et al., 2014; Li et al., 2014). Once bound, gabapentin inhibits the post-Golgi forward trafficking of the subunit to the surface of the cell, thus reducing VGCC expression (Zoidis et al., 2005). However, the magnitude of this reduction varies by subject, with one study reporting as few as 32% of patients seeing successful pain relief. Such variance in VGCC reduction suggests a possible reason for gabapentin's low overall efficacy as well as its success in attenuating nociceptive pain in certain patients (Li et al., 2014).

In successful cases, the downregulated VGCC activity and reduced excitatory glutamate release led to decreased activity of the 4-isoxazolepropionic acid (AMPA) receptor and decreased norepinephrine release within the brain (Chang et al., 2014). AMPA receptors facilitate synaptic transmission in the central nervous system and the locus coeruleus (LC). In the study conducted by Suto et al. this action on AMPA receptors was explored further using a mouse model after peripheral nerve injury. The researchers were particularly interested in the effect of gabapentin on the LC, suspecting the importance of the LC in activating descending inhibition. Specifically, the study found that neurons providing descending inhibition within this area were excited by gabapentin's action on astrocytes which increased glutamate tone within the LC. This increase in glutamate was induced via glutamate transporter-1 (GLT-1)-dependent mechanisms. Activation of the LC neurons was blocked when AMPA glutamate receptor antagonists were locally applied, confirming the importance of the AMPA receptor. This increase in glutamate was not seen within the spinal cord (Bao et al., 2014). Also of note, gabapentin was found to *decrease* GABA within the LC, a major site of descending pain inhibition, rather than the spinal dorsal horn (Pertovaara, 2006; Yoshizumi et al., 2012).

Several derivatives of gabapentin, including those with greater affinity for the α-2δ Ca^2+^ channels, have been used experimentally to support this method of action for the attenuation of neuropathic pain by gabapentin. N-type (Ca_v_2.2) VGCCs are of particular interest due to their role in transducing electrical activity into other cellular functions (Zoidis et al., 2005). An experiment involving the novel gabapentin derivative GABA adamantane (AdGABA) demonstrated AdGABA's efficacy in antagonizing pentylenetetrazole- and semicarbazide-induced tonic convulsions in addition to exhibiting analgesic activity in mice. As with gabapentin, AdGABA acts on the α-2δ subunit of voltage gated calcium channels, but with three-fold increased strength and affinity (Suto et al., 2014) AdGABA's increased affinity for this receptor and its downstream effects demonstrate the importance of the α-2δ subunit in pain processing and central sensitization.

A second derivative of gabapentin, 2-(aminomethyl) adamantane-1-carboxylic acid (GZ4), had similar effects on N-type Ca_v_2.2 channel currents, only in the presence of the α-2δ subunit (Zoidis et al., 2005; Suto et al., 2014). Administration of AdGABA and GZ4 resulted in the inhibition of excitatory neurotransmitters such as glutamate and reduced the presence of hyperalgesia and allodynia (Zoidis et al., 2005). These experiments demonstrate the importance of gabapentin's interactions with the α-2δ subunit of VGCCs in reducing hyperalgesia, however, this is not the only mechanism through which the drug exhibits its analgesic effect.

### Interleukin-10 and heme oxygenase-1 pathway

Recent studies have shown that the analgesic qualities of gabapentin may not be limited to the interaction at voltage gated calcium channels, but may also include interactions with the interleukin (IL)-10-heme oxygenase-1 (HO-1) signaling pathway. IL-10 is a key immunoregulatory cytokine with anti-inflammatory properties. The cytokine assists in regulating inflammation by suppressing the expression of pro-inflammatory cytokines, chemokines, and adhesion molecules, in addition to regulating antigen-presenting and co-stimulatory molecules in monocytes/macrophages, neutrophils, and T-cells. IL-10 induces heme oxygenase-1 in macrophages through an activated protein kinase cascade (Zoidis et al., 2014). The action of the heme oxygenase system is to induce the heme catabolic pathway. The heme catabolic pathway comprises of HO and biliverdin reductase, and generates products of heme degradation including carbon monoxide, iron, and biliverdin/bilirubin. The HO system plays an important role in controlling tissue homeostasis during inflammation by inhibiting pro-inflammatory cytokine synthesis and prompting anti-apoptotic processes (Asadullah et al., 2003; Zoidis et al., 2014). Several studies have shown the importance of the HO system in the attenuation of neuropathic pain. In a mouse model of sciatic nerve injury, carbon monoxide (CO)-releasing molecules and HO-inducing treatments have been shown to increase the anti-nociceptive effect of μ-opioids such as morphine in addition to inhibiting spinal microglial activation. CO-releasing and HO-inducing molecules were utilized due to their demonstrated ability to activate the cyclic guanosine monophosphate-PKG pathway, which is responsible for morphine's local antinociceptive effects (Abraham and Kappas, 2008). Gabapentin has been shown to positively influence interactions between both the IL-10 and HO-1 pathways (Compton et al., 2010; See Footnote 1).

Gabapentin used in conjunction with morphine has been shown to enhance the anti-nociceptive effect of IL-10 and HO-1 signal transduction pathway through inhibition of spinal inflammation in a preclinical neuropathic pain model. Significantly increased IL-10 levels were present with the co-administration of gabapentin and morphine as compared to morphine alone, supporting the role of gabapentin in this pathway. Furthermore, the use of anti-IL-10 antibody or zinc protoporphyrin, an HO-1 inhibitor, partially blocked the effect of gabapentin on morphine. These results implicate neuro-inflammation as a common mechanism in both neuropathy-induced and opioid-induced glial activation (See Footnote 1).

## Case reports of gabapentin efficacy

Although the mechanisms of gabapentin as well as opioid–induced hyperalgesia are not completely understood, the efficacy of gabapentin in attenuating OIH has been documented in several case studies. Compton et al. evaluated OIH and the effects of gabapentin in relieving experimental pain in methadone maintained patients. A 2400 mg PO dose was administered daily over a 1-week period, and pain was evaluated using a standardized CP test. The experimental group showed statistically significant improvements in pain threshold and tolerance compared to the control group at peak as well as trough methadone levels. The study concluded that when used in clinically tolerated doses, gabapentin significantly improved CP pain responses in methadone-maintained patients (Compton et al., 2010). This experiment provided evidence supporting the use of gabapentin in treating OIH.

Another study by Cuignet et al. evaluated gabapentin's ability to reduce OIH in burn patients receiving opioids. Patients were treated with 800 mg of gabapentin three times per day for 21 days. Pain levels were evaluated using a visual analog scale. In the earlier stages of the study, both experimental and control groups had similar pain scores. However, during the rest of the treatment phase, the pain scores became significantly smaller in the gabapentin group as did the required opioid dosage (Hervera et al., 2012). A reduction in opioid dosage with the use of gabapentin would also mean a reduced risk of developing OIH and allodynia.

Hauer et al. evaluated the effect of gabapentin in two separate cases of infants suffering from neurological impairment resulting from an injury to the central nervous system accompanied and possibly sustained by significant pain. These case reports identified pain sources as nociceptive, peripheral neuropathic, central neuropathic, and visceral hyperalgesic in nature. Significant improvement in apnea following empiric treatment with gabapentin was observed. In both cases, the initial gabapentin dose was 2.5 mg/kg with titration occurring every 4–7 days with the option to titrate more rapidly once dosage tolerance was confirmed (Cuignet et al., 2007). The reports proved gabapentin's role in the treatment and attenuation of hyperalgesia under altered neurologic states and amongst different age groups.

## Conclusion

OIH will remain an important issue so long as chronic opioid usage remains prevalent in pain control. As such, clinicians should exercise caution in prescribing opioids, and may consider prescribing alternative therapies for pain relief if available. As discussed in prior studies, the use of adjunctive therapies such as gabapentin may be applicable if OIH is suspected. Gabapentin strongly binds to the α-2δ subunit of dorsal horn VGCCs, downregulating their activity and diminishing the propagation of pain signals along afferent neurons. Gabapentin also leads to increased IL-10 and HO-1 pathway activation, promoting anti-inflammatory activity at sites of spinal neuron insult. At this time, the efficacy of gabapentin in mitigating OIH has been demonstrated in animal models and some human case studies. However, few large scale standardized patient studies have been performed to corroborate these findings. We propose the design and implementation of standardized studies investigating gabapentin use in pain patients receiving opioid medications to further elucidate mechanisms underlying OIH as well as to establish a statistical basis for gabapentin use as an adjuvant therapy.

### Conflict of interest statement

The authors declare that the research was conducted in the absence of any commercial or financial relationships that could be construed as a potential conflict of interest.

## Footnotes

^1^http://www.accessdata.fda.gov/drugsatfda_docs/nda/2003/075350_ORIGINAL%20APPROVAL_PACKAGE.pdf
